# Predicting Bone Metastasis Using Gene Expression-Based Machine Learning Models

**DOI:** 10.3389/fgene.2021.771092

**Published:** 2021-11-10

**Authors:** Somayah Albaradei, Mahmut Uludag, Maha A. Thafar, Takashi Gojobori, Magbubah Essack, Xin Gao

**Affiliations:** ^1^ Computer, Electrical and Mathematical Sciences and Engineering Division (CEMSE), Computational Bioscience Research Center (CBRC), King Abdullah University of Science and Technology (KAUST), Thuwal, Saudi Arabia; ^2^ Faculty of Computing and Information Technology, King Abdulaziz University, Jeddah, Saudi Arabia; ^3^ College of Computers and Information Technology, Taif University, Taif, Saudi Arabia

**Keywords:** metastasis, bone, gene experession, machine learining, hub genes, genetic diagnostic tool, deep learning

## Abstract

Bone is the most common site of distant metastasis from malignant tumors, with the highest prevalence observed in breast and prostate cancers. Such bone metastases (BM) cause many painful skeletal-related events, such as severe bone pain, pathological fractures, spinal cord compression, and hypercalcemia, with adverse effects on life quality. Many bone-targeting agents developed based on the current understanding of BM onset’s molecular mechanisms dull these adverse effects. However, only a few studies investigated potential predictors of high risk for developing BM, despite such knowledge being critical for early interventions to prevent or delay BM. This work proposes a computational network-based pipeline that incorporates a ML/DL component to predict BM development. Based on the proposed pipeline we constructed several machine learning models. The deep neural network (DNN) model exhibited the highest prediction accuracy (AUC of 92.11%) using the top 34 featured genes ranked by betweenness centrality scores. We further used an entirely separate, “external” TCGA dataset to evaluate the robustness of this DNN model and achieved sensitivity of 85%, specificity of 80%, positive predictive value of 78.10%, negative predictive value of 80%, and AUC of 85.78%. The result shows the models’ way of learning allowed it to zoom in on the featured genes that provide the added benefit of the model displaying generic capabilities, that is, to predict BM for samples from different primary sites. Furthermore, existing experimental evidence provides confidence that about 50% of the 34 hub genes have BM-related functionality, which suggests that these common genetic markers provide vital insight about BM drivers. These findings may prompt the transformation of such a method into an artificial intelligence (AI) diagnostic tool and direct us towards mechanisms that underlie metastasis to bone events.

## Introduction

Cancer-related morbidity and mortality are primarily associated with metastasis, and the most frequent site for tumor metastasis is the bone, particularly for breast and prostate cancers (Coleman, 1997; Landemaine et al., 2008). Also, cancer cells present in the bone marrow called disseminated tumor cells (DTCs) were shown to correlate with increased risk of disease recurrence and poor prognosis in early breast cancer (BCa) patients (Braun et al., 2005; Bidard et al., 2008). We now know that cancer metastasizing to the bone (BM), called osteotropism, requires stepwise processes that include tumor cells acquiring specific molecular characteristics to one/detach from the primary tumor, two/enter the bone, and three/home within the bone niche. However, the molecular pathways of metastases are still unknown despite the substantial advancements made in cancer-related therapies. Moreover, adjuvant treatment with bisphosphonates or denosumab only benefits specific patient subgroups (Paterson et al., 2012; Gnant et al., 2015; Jacobs et al., 2015). Thus, a number of groups have been attempting to unravel BM mechanisms using molecular biology methods (Kingsley et al., 2007).

Recent works (Josefsson et al., 2018; Rizzo et al., 2019; Pantano et al., 2020) used circulating tumor cells’ protein or gene expression profiles to suggest biomarkers for predicting BM. However, primary tumors’ protein or gene expression profiles are more commonly studied and recommended biomarkers for predicting BM. For example, high or elevated levels of CAPG, GIPC1 (Westbrook et al., 2016), ITGBL1 (Li et al., 2015), IL-1B (Li et al., 2015), DOCK-4 (Westbrook et al., 2019), nPAK4 (Li Y. et al., 2019), PRDX4 (Tiedemann et al., 2019), LPC1 (Tiedemann et al., 2019), and PRL (Sutherland et al., 2016) are all suggested BM biomarkers based on different studies. Also, several works (Kang et al., 2003; Smid et al., 2006; Sanz-Pamplona et al., 2012; Dean-Colomb et al., 2013; Zhou and Liu, 2014) have attempted to identify panels of BM-related genes from gene expression data. Few studies, such as (Smid et al., 2006; Zhou and Liu, 2014), used the identified genes as signatures to construct a model for predicting BM risk in breast cancer. Developing more such models that can predict BM from a disease specific and generic perspective with high performance accuracy could be used to support the physician’s work. Additionally, exploring the mechanism of BM from different primary sites and determining if this mechanism has common features despite originating from various primary sites is necessary, as it may provide a better understanding of the biological underpinnings of BM (Albaradei et al., 2021b).

In this study we performed a meta-analysis of three breast cancer and two prostate cancer gene expression profiles, to identify metastasis-related genes common to both cancer types. We started this process by identifying the differentially expressed genes (DEGs) between primary and metastasized tumors, then used these genes to construct a protein-protein interaction (PPI) network. We then calculated betweenness centrality (BC) to determine the hub genes which we used as input to develop machine learning models that can predict BM with high prediction accuracy. We developed support vector machine (SVM), random forest (RF), and deep learning network (DNN) models. The DNN model produced the highest prediction accuracy using only 34 top-ranked hub genes. Next, the robustness of the DNN model was validated using independent datasets from the cancer genome atlas (TCGA) and the metastasis-related functionality of the 34 top-ranked hub genes were validated by experimental evidence in existing literature.

## Method and Materials

### Gene Expression Datasets

We searched for gene expression datasets in Gene Expression Omnibus (GEO) (Edgar et al., 2002) using the following query: "metastas* AND bone AND Homo sapiens” filtered by “Expression profiling by array” on July 19th, 2021. We retrieved 241 entries that we sifted through but only found breast or prostate cancer samples with microarray gene expression data for primary tumors (without metastasis) and tumors with BM (metastasis to bone). The data used in this study include breast cancer data (GSE103357, GSE137842, GSE 2034) and prostate cancer data (GSE32269, GSE43332) (see Table 1). We fed this data to the ImaGEO tool (Toro-Domínguez et al., 2019) to perform the initial differential expression analysis, including background correction, normalization, and batch effect correction.

**TABLE 1:** Information of the gene expression datasets from GEO.

GEO accession	Platform	Total number of samples included	Metastasis	Non-metastasis
GSE103357	GPL6947	5	3	2
GSE137842	GPL570	6	3	3
GSE2034	GPL96	286	69	217
GSE32269	GPL96	51	22	29
GSE43332	GPL6244	14	8	6

### Meta-Analysis of Gene Expression Data

We used ImaGEO software, with default settings and the effect size method for the gene expression data meta-analysis. The tool transforms expression values to the logarithmic scale where needed, annotates the probe identifiers with unique Entrez Gene identifiers, merges the data, and provides data quality control checking. The tool further computes median values for duplicate gene expression profiles in each dataset, filters out genes with missing values in more than 10% of samples, and imputes missing values for the remaining genes using the average expression values in the respective primary or metastasis group.

We identified the DEGs using MetaDE.ES in the MetaDE package. This method tested the heterogeneity of gene expression value using three statistical parameters: τ^2^, Q-value, and Qpval. Then, we tested for differential expression of genes between the primary and metastasized groups using *p*-value. To ensure the homogeneity of featured genes, τ^2^ = 0, Qpval >0.05, and *p* < 0.05 were set as the cut-offs. The criteria for DEGs were false discovery rate (FDR) *p*-value < 0.05 and log2fold change >2. Thus, the MetaDE package performs heterogeneity tests first to determine if genuine differences underlie the results of the studies (heterogeneity) as opposed to variation based on chance alone, then selects DEGs successively (Wei et al., 2018), unlike commonly used limma, which selects DEGs based on *p*-value and fold-change thresholds.

### Constructing the PPI Network and Identifying Hub Genes

Many recent studies use GeneMANIA (Gene Ontology molecular function-based weighting) to analyze the gene lists and prioritize genes for functional assays (Taye et al., 2017). The reason being, it offers several advantages over other PPI networks in terms of flexibility, data representation, and predictive accuracy as it is a collection of many datasets and different interactions from GEO, BioGRID (Stark et al., 2006), IRefIndex (Razick et al., 2008), and I2D (Brown and Jurisica, 2007). Thus, we used the GeneMANIA Cytoscape 3.6.0 plugin (Montojo et al., 2014) to generate a physical protein-protein interaction network using the 534 DEGs. Briefly, we uploaded our 534 DEGs to Cytoscape, then selected the physical interactions option and removed the nodes with no connections. Next, we used the Cytoscape CytoHubba plugin to identify hub genes in the constructed PPI network via the BC scoring technique. Genes/proteins were ranked based on the BC score. DEGs among the top 100 hub genes were shortlisted and subsequently used to develop ML/DL models that distinguish between primary and metastasized samples.

### Using the Hub Genes as Features to Develop ML/DL Models

We created a parameter search space to evaluate different configurations for the SVM, RF, and DNN models (see Table 2). We implemented the SVM SVC class from the Scikit-learn Python library (Pedregosa et al., 2011). We employed the standard parameters, radial basis function kernel with degree = 3 and gamma = auto. We also implemented an RF model from the Scikit-learn Python library with 100 trees in the forest and max depth = 2. Also, we implemented DNN, a neural network with two hidden layers with 12 and eight nodes using the Python Keras library (https://github.com/fchollet/keras). We employed the SGD algorithm with the default parameters as the optimizer and used cross-entropy to compute the loss between actual and predicted labels. We set the number of epochs to 500 and the batch size to 8. We used the early stopping technique and the dropout technique with a drop rate of 0.3 to avoid overfitting. Because the number of samples is imbalanced, we also used the synthetic minority oversampling technique (SMOTE) to oversample the minority class using the imbalanced-learn python library (Chawla et al., 2002).

**TABLE 2:** Parameter search space for optimizing SVM, RF, and DNN models (Bold fond indicates the selected value).

Model	Parameter	Range
**SVM**	gamma	(“scale”, “auto”)
	Kernel	(“linear”, “poly”, “rbf”, “sigmoid”, “precomputed”)
**RF**	n_estimators	(1, 2, 4, 8, 16, 32, 64, 100,200)
	max_depth	(1, 2, 4, 8, 16, 32, 64, 100)
**DNN**	node size in each layer	(4, 8, 12, 16, 32, 64)
	activation function	(**“**relu**”**, “tanh”, “sigmoid”, “linear”)
	Optimizers	(SGD, “Adam”, “Nadam”]
	batch size	(4, 8, 16, 32)

We previously developed ML/DL models that successfully distinguish between primary and metastasis samples (Albaradei et al., 2019; Albaradei et al., 2021a). Thus, we here too iteratively added ten top-ranked genes based upon their BC value to train SVM, RF, and DNN models to mine the top essential genes that distinguish the primary and BM tumors.

We used the GEO integrated datasets (samples) for model training and computed the area under the curve (AUC) to evaluate the prediction performance of all the models. Using stratified random sampling technique (Pedregosa et al., 2011), we split the data into 80% training (296 samples) and 20% validation (74 samples). In addition, we used external testing data from the TCGA datasets to test the robustness of the best-performing model. The external set was extracted from the human cancer metastasis database (HCMDB) (Zheng et al., 2018), where we found 117 samples in which 38 were metastasized to bone (see the complete list of TCGA IDs in Supplementary Table S1). We computed sensitivity (Se), specificity (Sp), positive predictive value (PPV), negative predictive value (NPV), and AUC to evaluate the model on the test set.

### Validating the Metastasis-Related Functionality

To validate the metastasis-related functionality of the 34 featured hub genes, we conducted a literature review and used the R package to explore the diseases associated with the 34 featured genes based on the disease gene network (DisGeNet). The enrichment significance was calculated using gene set enrichment analysis (GSEA), a computational method determining if a predefined set of genes exhibit a statistically significant or concordant difference between two biological states (Subramanian et al., 2005).

## Results

### Study Design

**FIGURE 1:**
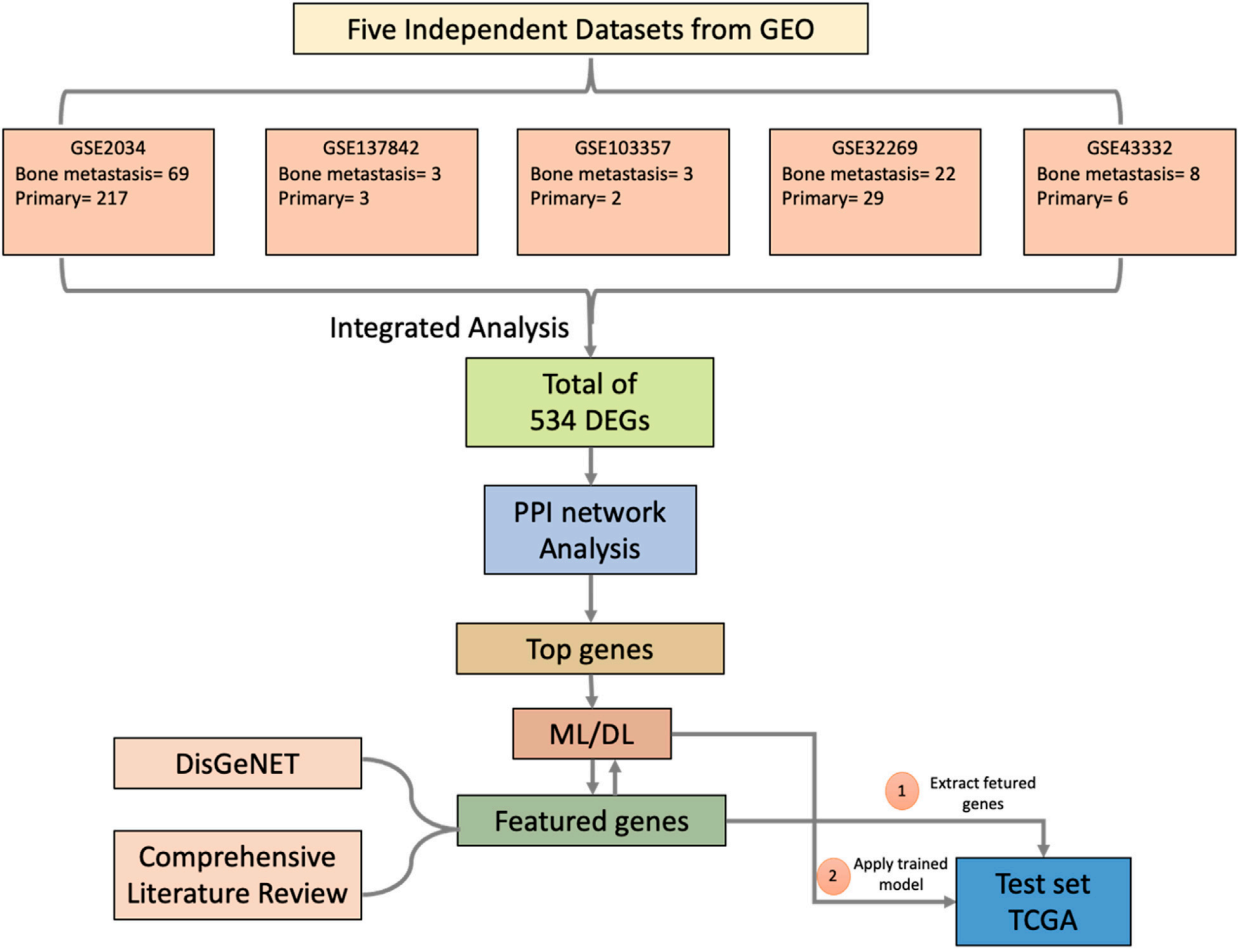
A Flowchart description of the study design.

The study design comprises six steps, depicted in a flowchart in Figure 1. First, we used ImaGEO to integrate and analyze the five GEO datasets and obtain DEGs (Step 1). Then, the DEGs were used to construct a gene-gene functional interaction network in GeneMANIA (Step 2). Next, we calculated network nodes’ betweenness centrality and degree centrality to determine the hub genes (Step 3). We then used the hub genes to develop ML/DL models that distinguish primary from metastasized samples (Step 4). Next, we validated the best-performing model using an independent test set from TCGA (Step 5). Finally, we conducted a literature review to validate the metastasis-related functionality of the 34 hub genes (Step 6).

### Differentially Expressed Genes (DEGs) Between Primary and Bone Metastasized Tumours

**FIGURE 2:**
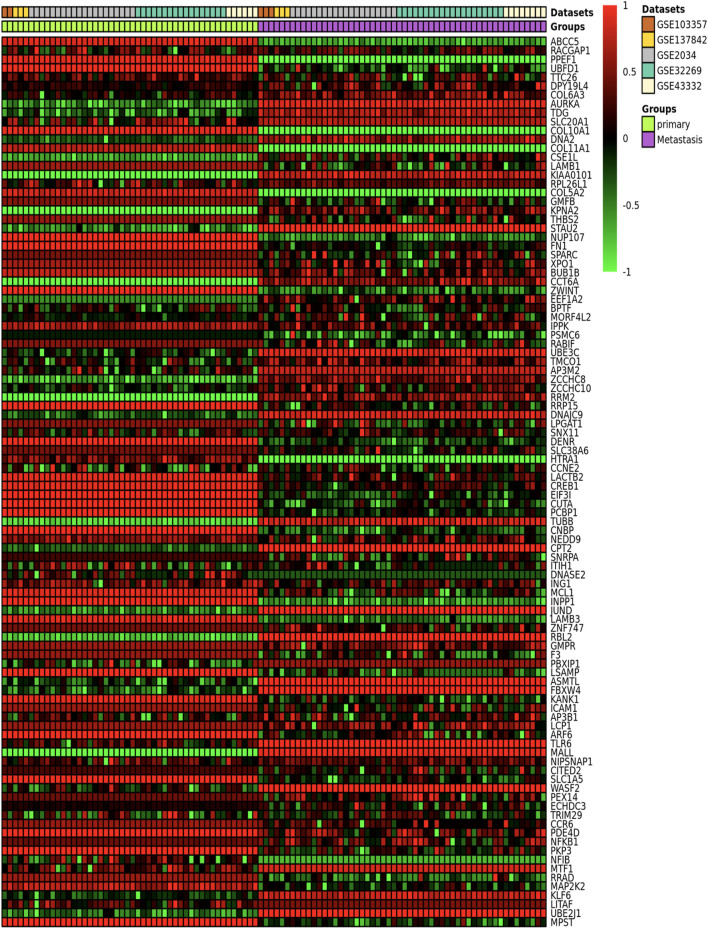
An ImaGEO generated heatmap of the top 100 DEGs based on the five microarray profiles.

Table 3 provides the ImaGEO tool’s quality control test results for the five gene expression datasets. The test shows the data used in this study is of good quality. The ImaGEO tool further annotated the probes with gene identifiers, merged and normalized data to provide the DEGs. The tool identified 534 DEGs, which include 365 up-regulated DEGs and 170 down-regulated DEGs. We provide the complete list of DEGs in Supplementary Table S2. A visual representation of the top 100 DEGs in the form of a heatmap shows the expression of more of the genes in the primary group is consistent in all the samples compared to the metastasized group (see Figure 2). Also, about 25% of these clearly down-regulated genes in the primary group are consistently up-regulated in the metastasized samples.

**TABLE 3:** The dataset quality control results generated by the ImaGEO tool.

ID	Samples preQC	Samples postQC	Imputed genes	PASS_QC
GSE103357	5	5	0	Yes
GSE137842	6	6	0	Yes
GSE2034	286	286	0	Yes
GSE32269	51	51	0	Yes
GSE43332	145	14	0	Yes

### Determining which DEGs are Hub Genes

The previous step provides us with the 534 DEGs but does not provide a means to identify the genes with the most functional impact, i.e., the so-called “hub” genes. Hub genes, according to research, are nodes that are highly connected to other nodes and are responsible for the majority of diseases such as cancer (Wachi et al., 2005). To identify the hub genes, we generated a gene-gene functional interaction network using GeneMANIA. First, the GeneMANIA software generates an interactive functional association network, comprising 634 nodes (which include the 534 genes and genes added based on the guilt-by-association approach) and 3,024 edges representing only direct physical protein-protein interaction (see Table 4). Then, we removed all the genes with no connected edges, leaving a network with 549 nodes and 3,005 connections. Next, we used the Cytoscape cytoHubba plugin to estimate the topological parameters, specifically, the betweenness centrality. Based on the BC score, we found 80 genes/proteins from the 534 DEGs among the top 100 hub genes. These 80 genes/proteins were subsequently used to develop ML/DL models that distinguish between primary and metastasized samples.

**TABLE 4:** The 80 DEGs ranked among the top 100 hub genes.

Rank	Hub genes	BC	Rank	Hub genes	BC	Rank	Hub genes	BC	Rank	Hub genes	BC
1	UBC	245,916.53	21	HSPA5	1094.87	41	TDG	292.11	61	CENPE	101.76
2	FN1	7,078.61	22	TNNT1	1094	42	GORASP2	251.35	62	CCT6A	98.94
3	XPO1	5,525.37	23	TUBB	863.24	43	WASF2	248.53	63	CCNB1	98.66
4	PCNA	2,237.74	24	DOLPP1	689.82	44	MMP14	246.27	64	FANCG	93.63
5	YWHAE	1851.59	25	PMM1	609.2	45	SP110	242.55	65	UBFD1	92.57
6	CSNK2B	1828.92	26	DCUN1D1	597.11	46	CREB1	237.59	66	CCND1	89.44
7	ACTB	1649.7	27	GAPVD1	592.8	47	SPARC	232.73	67	SHFM1	89.11
8	CUL2	1612.17	28	SEC61B	590.25	48	PROCR	219.23	68	PIAS1	88.99
9	HNRNPA1	1595.04	29	AURKA	576.99	49	NDUFA4	184.02	69	RAE1	80.2
10	COL1A1	1494.98	30	EZH2	573.01	50	MYH9	167.71	70	DNMT3B	79.91
11	TUBA1A	1480.27	31	RASA1	547.88	51	BUB1B	149.02	71	EIF3D	77.52
12	ILK	1439.63	32	SATB2	503.98	52	NACA	140.35	72	PHF20L1	77.41
13	TMEM109	1313.23	33	JAK2	483.91	53	PAXIP1	137.01	73	PINK1	76.5
14	VAPA	1294.47	34	RPL26L1	481.36	54	RAN	126.37	74	EEF1A2	71.12
15	PCBP1	1240.18	35	TUBA1C	424.14	55	JUNB	125.6	75	KIF2C	70.24
16	BUB1	1222.72	36	TUBA1B	395.71	56	NFKB1	122.42	76	KIF11	68.14
17	COL5A1	1194.25	37	HSPA9	372.74	57	RBBP4	121.81	77	IPO7	68.12
18	PSMA7	1171.14	38	CUL4A	353.56	58	MAD2L1	120.84	78	ICAM1	60.64
19	SSR4	1110.56	39	PRKCQ	325.42	59	ARF6	115.1	79	TOP2A	60.24
20	SCRIB	1098.59	40	EIF3I	300.9	60	KPNA2	106.04	80	CLNS1A	53.59

### Evaluating if the Hub Genes Can Be Used to Develop Robust ML/DL Models that Distinguish Primary and Metastasized Tumours

**FIGURE 3:**
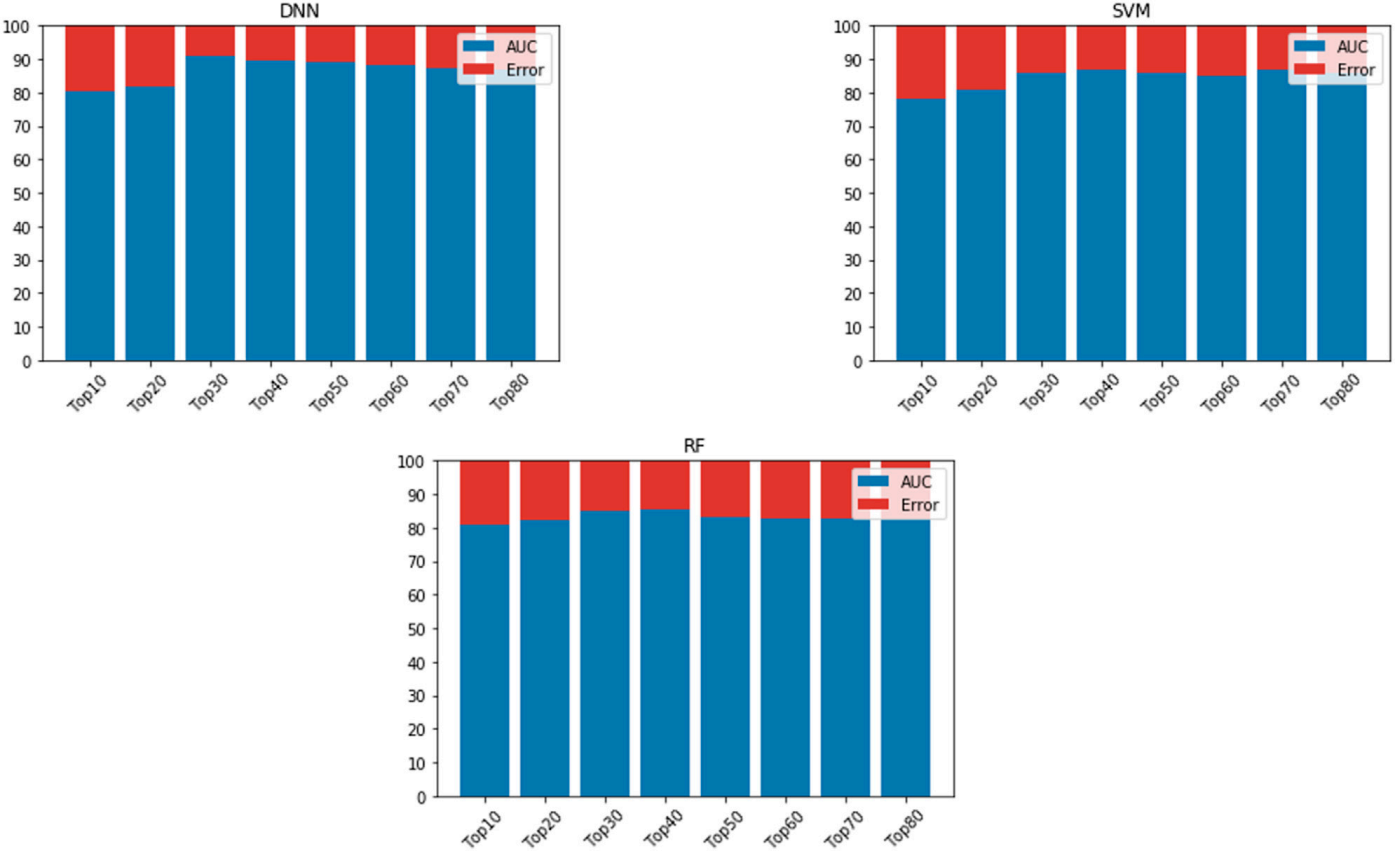
AUC is based on different numbers of featured genes using DNN, SVM, and RF. AUC is indicated in blue, while error rate is indicated in red.

We fed the 80 hub genes to each model (SVM, RF, and DNN) for training. That is, we iteratively added ten of the top-ranked genes based upon their BC value to train the models. The DNN model achieved the best AUC when including the 30 top-ranked genes (see Figure 3). We then evaluated the effect of adding some genes surrounding the 30 top-ranked genes to get the optimized performance. The 34 top-ranked featured genes (see Table 5) achieved the best performance with AUC of 92.11% and were selected to construct the final DNN model. To evaluate the robustness of this DNN model, we further used the model to distinguish primary and BM samples in a completely separate, “external” TCGA dataset. The DNN model achieved Se of 85%, Sp of 80%, PPV of 78.10%, NPV of 80%, and AUC of 85.78%. This result shows that the DNN model provides a more than satisfactory performance. Also, the models’ way of learning allowed it to zoom in on the featured genes that provide the added benefit of the model displaying generic capabilities in terms of the phenotype under investigation (primary versus BM).

**TABLE 5:** Metrics and literature linking the 34 feature genes to metastasis or specifically BM.

Genes	FDR	BC	Expression linked to metastasis	Linked to metastasis
UBC	0.038	245,916.531	down-regulated	n/a
FN1	0.001	7,078.614	up-regulated	Soikkeli et al. (2010)
XPO1	0.001	5,525.367	up-regulated	Gravina et al. (2014), Cotul et al. (2020)
PCNA	0.013	2,237.745	up-regulated	Zuo et al. (2020)
YWHAE	0.023	1,851.595	down-regulated	Leal et al. (2016)
CSNK2B	0.045	1,828.924	down-regulated	n/a
ACTB	0.013	1,649.700	down-regulated	n/a
CUL2	0.022	1,612.171	up-regulated	Meng et al. (2018)
HNRNPA1	0.039	1,595.038	up-regulated	Loh et al. (2015), Chen et al. (2018)
COL1A1	0.013	1,494.975	up-regulated	Liu et al. (2018a)
TUBA1A	0.039	1,480.275	up-regulated	n/a
ILK	0.015	1,439.632	down-regulated	Zhu, Liu et al. (2012)
TMEM109	0.042	1,313.235	down-regulated	n/a
VAPA	0.014	1,294.474	up-regulated	Zhang et al. (2020), Zhou et al. (2020)
PCBP1	0.006	1,240.178	down-regulated	Wang et al. (2010)
BUB1	0.014	1,222.716	up-regulated	n/a
COL5A1	0.005	1,194.251	up-regulated	Liu et al. (2018b), Feng et al. (2019)
PSMA7	0.049	1,171.135	up-regulated	n/a
SSR4	0.018	1,110.555	down-regulated	n/a
SCRIB	0.021	1,098.592	up-regulated	Hussein et al. (2021)
HSPA5	0.010	1,094.874	down-regulated	n/a
TNNT1	0.026	1,094.000	up-regulated	n/a
TUBB	0.010	863.241	down-regulated	n/a
DOLPP1	0.004	689.825	up-regulated	n/a
PMM1	0.046	609.200	down-regulated	n/a
DCUN1D1	0.038	597.108	up-regulated	Shuang et al. (2017), Li et al. (2019c)
GAPVD1	0.012	592.799	up-regulated	n/a
SEC61B	0.021	590.249	up-regulated	n/a
AURKA	0.000	576.994	up-regulated	Yang et al. (2016), Chen et al. (2017)
EZH2	0.004	573.010	up-regulated	Shin and Kim (2012), Hirukawa et al. (2018)
RASA1	0.047	547.878	up-regulated	n/a
SATB2	0.038	503.985	up-regulated	Seong et al. (2015), Luo et al. (2016)
JAK2	0.025	483.914	up-regulated	Talati et al. (2015), Wang et al. (2017)
RPL26L1	0.000	481.357	up-regulated	n/a

### Validating the Metastasis-Related Functionality of the 34 Top-Ranked Hub Genes

Thus far, the gene-gene functional interaction network allowed us to predict several of the critical metastasis-related genes based on diverse metrics, including FN1 with the lowest FDR value (0.001) and highest BC value (7,078.61), and XPO1 with a similarly low FDR value (0.001) and high BC value (5,525.37). Therefore, FN1 and XPO1 were the most important hub genes among DEGs across five microarray studies, followed by UBC (FDR 0.038, BC 245916.54), PCNA (FDR 0.0127, BC 2237.75), and YWHAE (FDR 0.0233, BC 1851.59).

**FIGURE 4:**
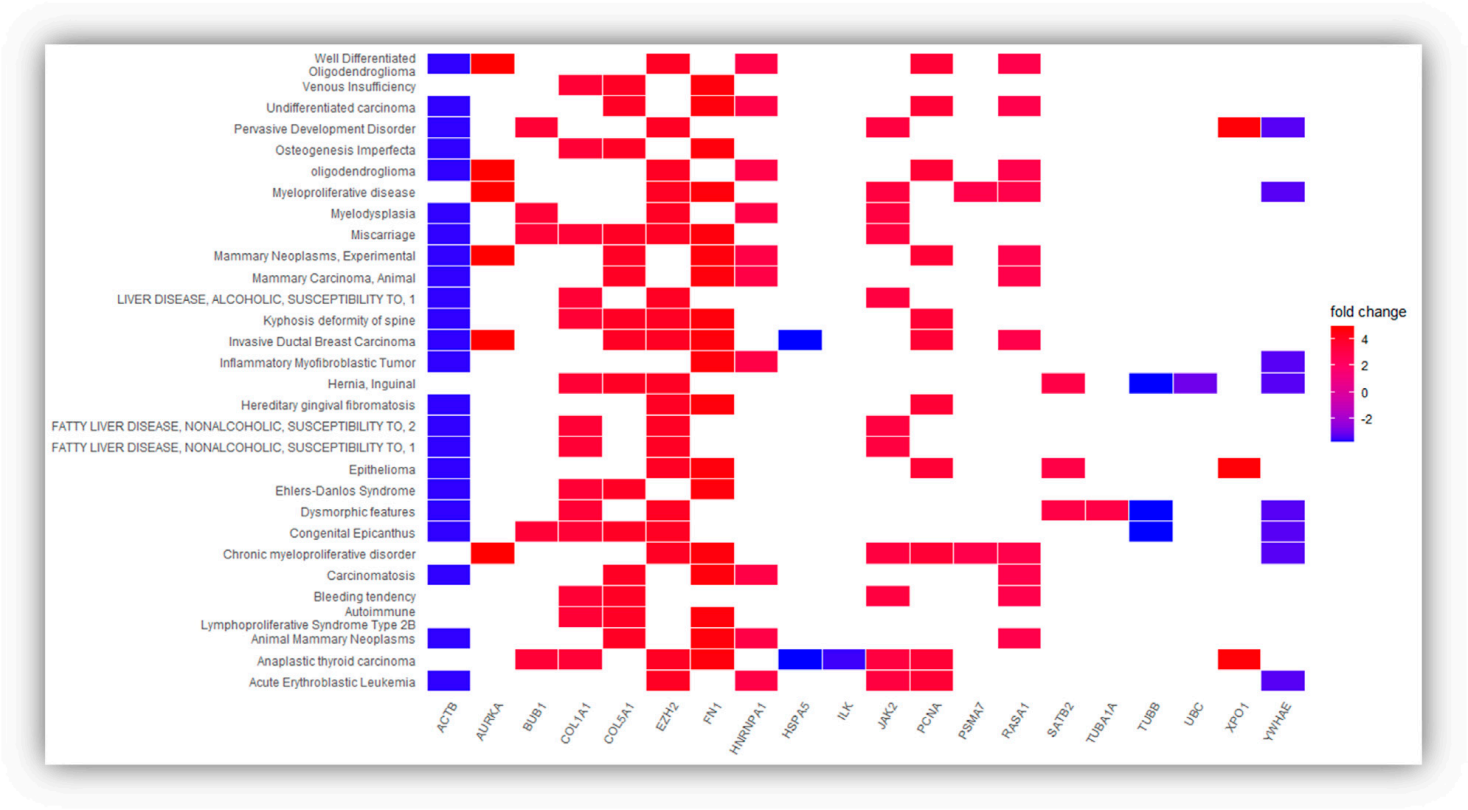
Represent the significantly over-expressed and under expressed genes present in the DisGeNET disease and genes involved in the significantly enriched DisGeNET disease. The depth of the color represents the fold change and the names of DisGeNET disease displayed vertically.

However, we still do not know the gene-disease associations of the 34 hub genes or if available experimental evidence links the genes to metastasis-related functionality. Thus, we evaluated the gene-disease associations of the 34 hub genes using DisGeNET (see Figure 4). DisGeNET indicates that the genes are associated with numerous types of cancer, autoimmunity, and bone disorders. For example, featured genes such as COL1A1, COL5A1, FN1, and ACTB are involved in invasive breast carcinoma and osteogenesis imperfecta, a heritable bone fragility disorder associated with short stature and abnormalities. This links these genes to breast cancer and bone softening, which is a feature of BM. In addition, genes such as COL1A1, HSPA5, FN1, ACTB, HNRNPA1, COL5A1, JAK2, and RASA1 are involved in Carcinomatosis and Mastitis, which shows these genes are involved in cancer spread throughout the body and inflammation in breast tissue. Also, FN1, PCNA, ACTB, COL1A1, EZH2, JAK2, and HSPA5 are involved in ureteric obstruction, an outcome of long-term invasive prostate cancer (Deng, Liu et al., 2015). This is interesting as a 2006 case report indicates that ureteric obstruction is a rare manifestation of metastatic breast cancer and that the obstruction may be due to retroperitoneal fibrosis, retroperitoneal or ureteric metastases. Furthermore, gastric cancer and renal cell carcinoma can also cause similar manifestations (Jani, 2006). We also conducted a literature review to provide a type of verification that the genes pinpointed in this study are indeed involved in metastasis-related functionality (see Table 5). As a result, we found literature supporting 17 of the 34 hub genes having known metastasis-related functionality. These results provide confidence that about 50% of the 34 hub genes have BM-related functionality and provide a birds-eye-view of the knowledge or lack of knowledge related to underlying BM mechanisms.

## Discussion

Certain types of cancer, such as breast and prostate, migrate to and grow in the bone microenvironment due to specific conditions. However, the number of large-scale gene expression research undertaken to identify the shared genetic markers responsible for BM is low. Therefore, this study aims to perform a meta-analysis of the primary site and BM-related gene expression profiles from breast and prostate cancers to identify BM-related genes common to both cancer types. First, we identified the DEGs and the subset of hub genes that we can use as features in the ML/DL models to distinguish between the primary tumors and the BM. Then, we tested how generic the best-performing model is with respect to predicting BM for samples from different primary sites, but could not compare our model related models because all previous works are based on predicting BM from one primary site. However, we could not compare our model to related models because all previous works predict BM from one primary site. Additionally, we are exploring BM from different primary sites to determine common features despite originating from various primary sites. Thus, this work is different from previous works. Nonetheless, the developed model predicts BM from a disease-specific and generic perspective with high-performance accuracy, which could support the physician’s work if transformed into an AI tool.

To recap, we set out to perform a BM-related meta-analysis across different cancer types but only found five GEO gene expression datasets associated with prostate and breast cancers that fulfil this criterion. Briefly, the methodology we implemented allowed us to identify 534 DEGs (*p*-value <0.05) shortlisted to a subset of 80 hub genes based on betweenness centrality. Next, we fed the 80 top-ranked hub genes as features to each machine learning model, including SVM, RF, DNN models. In this manner, we filtered the genes to prioritize the most significant hub genes based on AUC using ML/DL models. Then, to test the robustness of the best-performing model, we used an external set (Zheng et al., 2018) comprising 117 samples, of which 38 were metastasized to bone. The DNN model achieved Se of 85%, Sp of 80%, PPV of 78.10%, NPV of 80%, and AUC of 85.78%. These results provide a good indication of the potential power of the selected 34 featured genes combined with a DNN to predict BM for samples from different primary sites, promoting the development of artificial intelligence (AI) diagnostic tools to enhance BM treatment.

Beyond that, these findings point out key genes involved in the metastasis process. Specifically, we further validated that more than 50% of the 34 hub genes have metastasis-related functionality. Here we mention the metastasis-related functionality exhibited by the products of a few of the top-ranked hub genes. Soikkeli and others demonstrated that the transforming growth factor-β signaling pathway is activated during metastatic outgrowth, and transforming growth factor-β inducible genes, including POSTN, FN1, and COL-I and VCAN, are up-regulated (Soikkeli et al., 2010). Moreover, they showed that POSTN, FN1, VCAN, and pro-collagen-I (PCOL-I, newly synthesized COL-I) colocalize in extracellular strand and ring structures, visible inside the metastases and at the tumor-stroma interface. Later findings supported this work, as Li and others demonstrated that small interfering RNA (siRNA)-mediated downregulation of FN1 suppress the migration, invasion, adhesion, proliferation capabilities, and induced apoptosis of melanoma cells (Li B. et al., 2019). Additionally, Armstrong and others also demonstrated that depletion of fibronectin (FN1) by siRNA knockdown markedly reduce the invasive capacity of prostate cancer (PCa) cells (Armstrong et al., 2018). Then, we have Exportin 1 (XPO1), one of the few exportins involved in transporting several tumor suppressor proteins (including p53, BRCA1, Survivin, NPM, APC, and FOXO) out of the nucleus. Gravina and others used a selective inhibition of XPO1, Selinexor (KPT-330), to demonstrate that XPO1 inhibition affects the metastatic potential of PCa cells using one model of intraprostatic tumor growth and two models of bone metastasis (Gravina et al., 2014). Concerning PCNA, Cui and others demonstrated that small hairpin RNA(shRNA)-mediated knockdown of a nuclear effector of the Hippo pathway, Yes-associated protein 1 (YAP1), down-regulate the expression of AxI, PCNA, and MMP-9, and inhibit the proliferation and invasion of human lung adenocarcinomas and gastric adenocarcinoma cells (Cui et al., 2012). Also, Zuo and others wanted to examine the role of circ-SMAD7 in glioma progression (Zuo et al., 2020). They demonstrated that downregulated Circ-SMAD7 inhibits cell proliferation, migration, and invasion in glioma cells. In addition, repressed PCNA mRNA and protein expression was observed after circ-SMAD7 was knocked down in the glioma cells, suggesting circ-SMAD7 promotes proliferation and metastasis of glioma via upregulating PCNA. In another study, Meng and others aimed to investigate how the key epithelial-mesenchymal transition (EMT) protein, Twist 1, increases vimentin expression (Meng et al., 2018). They reported that Twist1 binds to the Cullin2 (Cul2) promoter to activate the selective transcription of Cul2 circular RNA (circ-10720), but not mRNA. The circ-10720 absorb miRNAs that target the vimentin, and it is in this indirect manner that Twist1 promoted vimentin expression. They further demonstrated that circ-10720 knockdown represses the tumor-promoting activity of Twist1 *in vitro* and patient-derived xenograft.

Overall, the experimental evidence shows that downregulation of several of the upregulated top-ranked hub genes suppresses the metastasis-related process, including migration, invasion, adhesion, and proliferation capabilities. Additionally, their functionality extends from being structurally-related to affecting the transportation of tumour suppressor genes and even eliminating miRNA that suppresses genes with metastasis functionality. Moreover, experimental evidence shows that silencing of the downregulated top-ranked hub genes such as YWHAE (Leal et al., 2016), ILK (Zhu et al., 2012) and PCBP1 (Wang et al., 2010) induces cell proliferation, migration, and/or invasion.

The present work yields the common genetic markers between breast and prostate cancer and provides vital insight about BM drivers. Additionally, more research focused on the subset of genes with no experimental evidence may yield new biomarkers or treatment targets.

## Concluding Remarks

To our knowledge, this is among the few studies to consolidate data on various cancer types, allowing us to understand the shared or consistent biological features of BM. In addition, this research unveiled several new and previously unknown genes related to BM. The last thing to mention is that, in this study, we developed a high-accuracy DNN model with 34 featured or hub genes. As far as we know, the primary site associated with BM does not hamper the models’ prediction performance. Therefore, we will focus our future work on identifying the unknown but “standard” molecular mechanisms that underlie BM from any primary site and transforming the model into an AI diagnostic tool.

### Availability

We also developed a web server to serve the scientific community. The web-based tool, bone metastasis predictor https://www.cbrc.kaust.edu.sa/bonemetastasis/, implements the DNN model developed in the current study to allow the users to predict the BM state of their sample using gene expression quantification values. The user needs to provide the gene expression of the genes for every sample. The number of samples corresponds to the number of rows in a file. The output includes a list of samples and indicates if the prediction is primary or BM.

## Data Availability

Publicly available datasets were analyzed in this study. This data can be found here: Gene Expression Omnibus (GSE103357; GSE137842; GSE 2034; GSE32269; GSE43332).
